# Live Yeast Supplementation Attenuates the Effects of Heat Stress in Dairy Cows

**DOI:** 10.3390/vetsci12090791

**Published:** 2025-08-22

**Authors:** Ana R. J. Cabrita, Júlio Carvalheira, António J. M. Fonseca

**Affiliations:** 1 REQUIMTE, LAQV, ICBAS, School of Medicine and Biomedical Sciences, University of Porto, Rua Jorge Viterbo Ferreira 228, 4050-313 Porto, Portugal; 2 CIBIO, ICBAS, School of Medicine and Biomedical Sciences, University of Porto, Rua Jorge Viterbo Ferreira 228, 4050-313 Porto, Portugal; jgc3@cibio.up.pt

**Keywords:** dairy cow, performance, temperature, thermal humidity indices, yeast

## Abstract

High temperature typically decreases feed intake, milk production, and efficiency and increases metabolic disorders and health problems, greatly impacting farm economics. Supplements based on *Saccharomyces cerevisiae* have been suggested to benefit cows under heat stress, but effects on dairy cow performance are contradictory. This study aimed to evaluate the influence of heat stress on the effects of live yeast supplementation on the performance of dairy cows. Environmental temperature parameters were compared to two thermal humidity indices (THI1 and THI2) using wet bulb or dew point temperatures, as explanatory variables of dairy cow performance during the hot season. The experiment followed a randomized complete block design with 12 Holstein cows blocked by lactation number, days in milk, and milk production (two cows per block) and within each block, each cow was randomly assigned to a maize silage-based TMR with a concentrate mixture containing no yeast culture (Control) or 1 g/kg concentrate dry matter of a live yeast culture based on *S. cerevisiae* (Yeast) for 35 days. The experiment lasted for 35 d. Dry matter intake (DMI) was significantly higher for Yeast than it was for Control for all classes of temperature and THIs studied with an average increase of 2 kg DM per day, except for mean THI1 (from 54 to 60), for which the DMI was similar between treatments. Yeast promoted significantly higher milk yield than Control for all classes of daily maximum and mean temperature, averaging an increase of 4 kg of milk per day. Results suggest a more marked effect of temperature and indicate that yeast supplementation improved lactation performance of dairy cows exposed to hot weather.

## Simple Summary

Supplements based on live yeast have been suggested to minimize negative effects of high temperature on dairy cow performance, but with conflicting results. This study evaluated the influence of heat stress on the effects of supplementing dairy cows’ diet with live yeast, by comparing environmental temperature parameters to two thermal humidity indices using wet bulb or dew point temperatures. Yeast supplementation improved the lactation performance of dairy cows exposed to hot weather with a more marked effect of temperature.

## 1. Introduction

Lactating dairy cows exposed to high ambient temperatures typically show decreased dry matter (DM) intake (DMI), milk production, and efficiency of milk yield, increased metabolic disorders and health problems, compromised milk quality, and decreased reproductive performance [1,2], with a great impact on farm economics [3,4]. Several thermal indices that combine different environmental factors have been proposed to measure the level of heat stress, but their use is still limited by lack of available on-farm data such as wind speed, rainfall, and thermal radiation. Data focused on environmental temperature and relative humidity, easily obtained from nearest meteorological stations to commercial barns, has been used by several studies [5,6,7,8]. Conversely to daily temperature, relative humidity is not commonly measured in commercial settings. Similarly, despite body temperature being an excellent indicator of the susceptibility of an animal to heat stress, devices for its measurement are not feasible for large number of animals [5]. Therefore, relationships between environmental temperature and dairy performance would be useful for evaluating effects of heat stress in practical situations. However, they do not allow the early identification of vulnerable animals that need more proactive interventions to decrease stress, thus maintaining performance. Recent and emerging approaches, such as analysis of fecal microbiome, cortisol measurements in feces, urine, hair, saliva, and milk, infrared thermography, remote sensing via rumen boluses and ear canal sensors, and machine learning analysis of sensor data, comprise tools that enable targeted mitigation strategies towards a sustainable dairy production under a climate change scenario [9].

The most comfortable environmental temperature range for dairy cattle is between 5 and 25 °C [10]. Dairy cows maintain internal stability through complex thermoregulatory mechanisms, relying on sensible and insensible heat loss to regulate body temperature. Key environmental contributors to heat stress include air temperature, humidity, wind, solar radiation, and rainfall, with temperature and relative humidity being especially critical. As recently reviewed by Singaravadivelan, Prasad [9], dairy cattle respond to heat stress with behavioral changes (panting, reduced activity, seeking shade), physiological adjustments (reduced feed intake, elevated respiration), and endocrine responses (cortisol and altered thyroid hormone levels). Additionally, cattle undergo acclimation and adaptation to cope with heat. The endocrine system, particularly the hypothalamic–pituitary–adrenal axis, plays a central role, adjusting hormone levels to reduce heat production and manage energy use. During extreme heat, evaporative cooling (through sweating and panting) is the main thermoregulatory mechanism, these processes being strongly influenced by temperature and humidity. Therefore, temperature–humidity indices are recommended as indicators for assessing heat stress in dairy cows.

Under conditions of high temperature and humidity, environmental and nutritional methods should be established to minimize the rise in body temperature and to improve the lactation performance of dairy cows [10,11,12]. As heat stress is known to adversely affect the function of the rumen microbiome and metabolism, namely increasing lactate and decreasing acetate producing bacteria [13], it is important to start ration adjustments before the onset of heat stress to allow time for rumen microflora to adapt. Although supplementation with fungal cultures, such as those based on *Saccharomyces cerevisiae*, has been suggested to benefit cows by reducing signs of heat stress, such as rectal temperature, and respiration rate [11], reported effects on dairy cow performance are contradictory. While some studies observed an increase in DMI [14,15] and milk production [11,16,17], others reported an absence of effects [16,18,19]. Suggested mechanisms of action of live yeast include oxygen scavenging in the rumen that reduces rumen redox and stimulate the growth of certain microorganisms [20], namely those that use lactic acid as substrate and population of cellulolytic bacteria [21], thus decreasing lactate accumulation in the rumen, stabilizing ruminal pH, and benefiting fiber digestion [22]. Increased ruminal pH with yeast supplementation has also been attributed to a reduction in the rate of starch digestion in the rumen [23,24], increased meal frequency, smaller meal sizes, and longer periods of rumination [25,26] that might deliver more saliva to the rumen, increasing buffering capacity, favoring fiber digestion, and reducing lactate concentration after a meal [22,27]. Moreover, yeast supplementation has been shown to attenuate the risk of inflammatory insults and the subsequent acute-phase response, thus preserving nutrients otherwise used to resolve inflammation [22,23,25]. The beneficial effects of yeast products appear to depend on days in milk (DIM; (DIM; [28])), level of yeast supplementation [29], yeast type [18], diet characteristics [30], and environmental conditions [31].

The present study aims to test the hypothesis that live yeast supplementation attenuates the negative effects of heat stress on dairy cow performance. It also aims to evaluate the relative effectiveness of different environmental temperature parameters and thermal humidity indices (THIs) in explaining the variations in performance during high-temperature periods. Understanding these relationships is increasingly relevant for optimizing dairy cow management strategies to mitigate heat stress and to improve the resilience of dairy production systems in a climate change scenario with more frequent sudden heat waves in unusual seasons of the year.

## 2. Materials and Methods

### 2.1. Animals, Housing, and Feeding

Twelve Holstein cows (parity number 2.5 ± 1.93) averaging a 600 ± 73.1 kg body weight, 115 ± 66.0 DIM, and 33 ± 10.4 kg of milk/day from the Dairy Unit of the Direção Regional de Agricultura do Entre-Douro e Minho (DRAEDM) of the Ministry of Agriculture (Paços de Ferreira, Portugal) were used. Sample size was estimated using the resource equation, commonly applicable to animal experiments, which calculates the degree of freedom of analysis of variance (E value) as the difference between the total number of animals and the total number of experimental treatments. The E value of the present study, in compliance with ethical issues, is 10, within the recommended range of 10 to 20 [32]. The experiment was a randomized block design with cows blocked by lactation number, DIM, and milk production and, within each block, randomly assigned to one of two treatments. This approach minimized possible errors due to the reduced number of animals per block (2 animals per block) and treatment (6 animals per treatment). Treatments were a diet comprising (DM basis) 420 g/kg of maize silage, 80 g/kg of coarsely chopped ryegrass hay, and 500 g/kg of concentrate mixture containing no yeast culture (Control) or 1 g/kg of a live yeast culture based on *S. cerevisiae* strain 1026 (Yeast; Yea-Sacc 1026, Alltech, Sintra, Portugal; Commission Regulation (EC) No. 1811/2005; Table 1). According to the manufacturer’s information, the minimum quantity of yeast given by 1 g of product is 2.5 × 108 cfu.

The experimental period lasted for 35 d. During 1 wk before starting the trial, cows were fed the Control diet and individual feed intake, milk production, and composition were recorded to be later used as covariables. This 1 wk period was considered sufficient as the control diet used comprised the same forages and similar a forage/concentrate ratio when compared to the previous diet offered to the cows, and the farm management was maintained during the experiment. Animals were housed and individually fed in a tie-stall barn and had continuous access to water. Diets were mixed as a TMR and offered for ad libitum intake, twice daily, at 08:30 and 16:30 h. The troughs were cleaned out each morning, and orts were collected and weighed throughout the experiment. Samples of maize silage, ryegrass hay, concentrates, and orts were sampled 3 times/week on non-consecutive days and, after oven DM determination, were composited for chemical analysis. Feed offered was adjusted each week to produce weigh backs of about 10% of amounts fed.

### 2.2. Measurements

Cows were milked twice daily at 07:00 and 17:00 h. Milk production was measured throughout the experimental period. Milk was sampled 3 times/week at both milkings for analysis of fat, protein, and lactose ([33]; Milkoscan 133, Foss Electric, Hillerød, Denmark]).

Meteorological data were obtained from the nearest weather station (Santo Tirso, Portugal) and consisted of daily maximum, minimum, and mean temperatures and relative humidity. Daily thermal amplitude was calculated as the daily difference between maximum and minimum temperatures. Wet bulb temperature (Twb), the lowest temperature an object may be cooled to by evaporation, was obtained from the calculator provided by Mountain View Technologies (Berkshire, UK), and dew point temperature (Tdp), the temperature at which water vapor starts to condense out of the air, was calculated from ambient temperature and relative humidity using the software provided by Colorado State University (Fort Collins, CO, USA). Two THIs empirically determined in cattle exposed to heat stress conditions in climatic chambers were compared in this study, all temperature values being in degrees Celsius: THI1 = (0.35 × Tdb + 0.65 × Twb) × 1.8 + 32 [34]; THI2 = (Tdb + 0.36 × Tdp) + 41.2 [35]. Daily maximum, mean, and minimum temperatures were used for calculating the maximum, mean, and minimum THIs, respectively.

### 2.3. Chemical Analysis

Samples of maize silage, ryegrass hay, concentrate mixtures, and orts were dried at 65 °C for 48 h in an air-circulating oven. Dried samples were then ground to pass through a 1 mm screen. Composited samples were analyzed for ash ([33]; method 942.05]) and Kjeldahl N ([33]; method 954.01). Crude protein (CP) was calculated as Kjeldahl N × 6.25. Neutral detergent fiber (NDF), acid detergent fiber (ADF), and acid detergent lignin (ADL) were determined by the procedures of [36,37], with heat-stable α-amylase being added, except for ryegrass hay, during NDF extraction; sodium sulfite was not added. Neutral detergent fiber was expressed without residual ash. Ether extract (EE) was determined by extracting the sample with petroleum ether using an automatic Soxhlet extractor (Gerhardt Analytical Systems, Königswinter, Germany). Phosphorus and Ca were determined by official Portuguese standard methods [38,39]. Starch and urea were analyzed using finely ground samples (0.5 mm screen) using the method described by [40] and by the official Portuguese standard method [41], respectively.

### 2.4. Statistical Analysis

The experiment was designed according to a completely randomized block design over 35 days, with two dietary treatments randomly distributed across six blocks. During the 7-day preliminary period, the animals were fed the control diet to collect baseline data on feed intake, milk production, and milk composition.

To assess the primary effects of diet and its interactions with environmental conditions, a mixed model was designed. Environmental parameters (daily temperature or THI) were incorporated as covariates in the model to estimate the regression coefficients within the diet (linear, quadratic, and cubic, as appropriate). Preliminary period variables (DMI, milk yield, milk protein, fat, and lactose content and production) were also included in the model as covariates. Daily data were analyzed using the Mixed procedure of SAS^®^ OnDemand for Academics (SAS Institute Inc., 2025), according to the following model:

$$y=\mu +bl+di+\left[\sum_{k=1}^{3}\left(bx\right)\right]di+cow\left(di\right)+prelim+e$$

where

µ = overall mean;
bl = main effect of block;
di = main effect of diet;
b = regression coefficients of daily temperature or THI within diet;
x = daily temperature or THI covariates within diet;
cow(di) = random effect of cow nested within diet;
prelim = preliminary period covariables;
e = random residual error, assuming N ~ (0, σ^2^_e_).

When the environmental and preliminary covariates were not significant (*p* > 0.05), they were dropped from the model. Differences between levels of main effects with *p* ≤ 0.05 were considered significant, whereas tendencies toward differences were accepted if 0.05 ≤ *p* ≤ 0.10. Models were compared using the likelihood ratio test (LRT). Diet differences between means of predicted values within environmental parameter classes (3 to 6 classes defined according to the parameter being compared) were analyzed using Student’s t-test. The environmental parameter classes were defined as follows:

Temperature (°C):

─ Minimum: 9–14, 14–19, 19–24, 24–29;
─ Mean: 16–19, 19–22, 22–25, 25–28, 28–31;
─ Maximum: 20–23, 23–26, 26–29, 29–32;
─ Amplitude: 8–10, 10–12, 12–14, 14–16, 16–18, 18–20.

THI1 (%):

─ Minimum: 54–60, 60–66, 66–72, 72–78;
─ Mean: 54–60, 60–66, 66–72, 72–78, 78–84;
─ Maximum: 60–66, 66–72, 72–78, 78–84, 84–90.

THI2 (%):

─ Minimum: 54–60, 60–66, 66–72, 72–78;
─ Mean: 60–66, 66–72, 72–78;
─ Maximum: 66–72, 72–78, 78–84, 84–90.

## 3. Results

**Figure 1:**
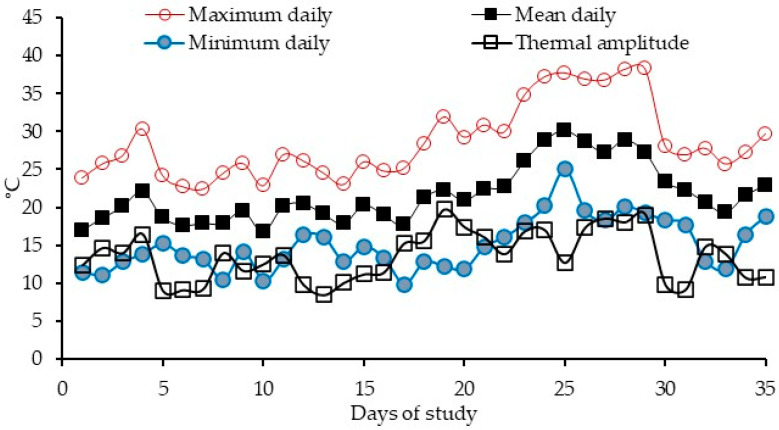
Daily thermal amplitude (calculated as the difference between maximum and minimum temperatures) and maximum, minimum, and mean temperatures during the study period.

**Figure 2:**
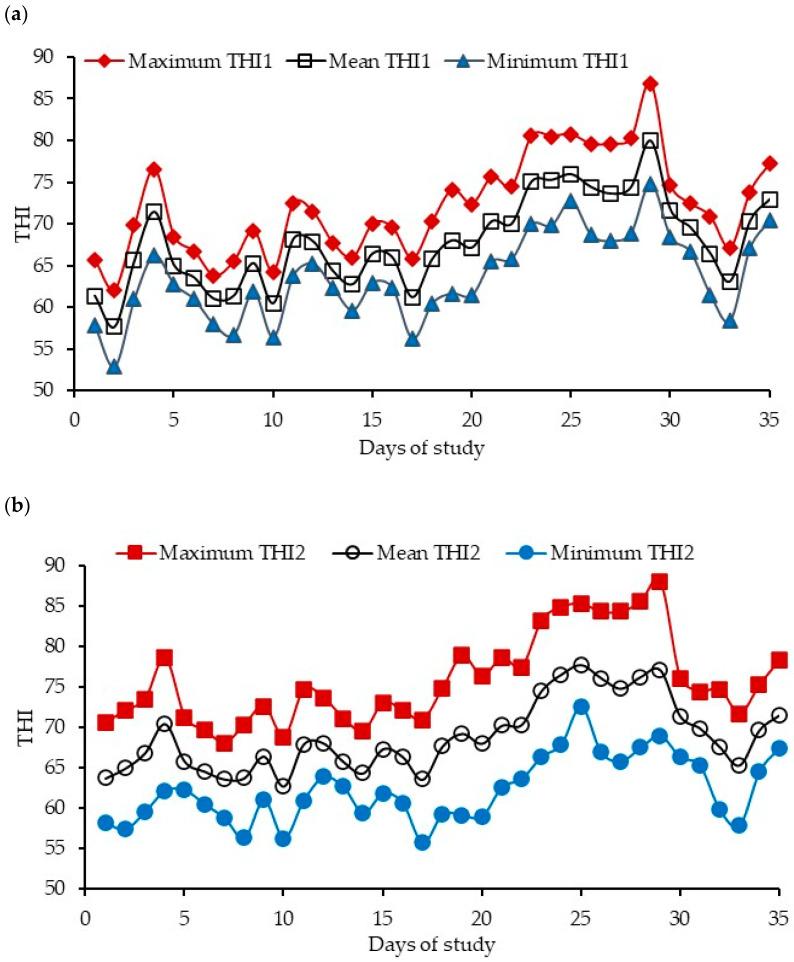
Daily maximum, mean, and minimum thermal humidity indices calculated according to (**a**) Bianca ([34]; THI1) and (**b**) Yousef ([35]; THI2) during the study period.

The maximum, minimum, and mean daily temperatures during the study period averaged 29 ± 4.9 °C, 15 ± 3.5 °C, and 22 ± 3.8 °C, respectively (Figure 1). The daily relative humidity, maximum, mean, and minimum THI1 and THI2 averaged 74 ± 11.6%, 72 ± 5.9, 68 ± 5.3, 64 ± 5.1, 76 ± 5.5, 69 ± 4.4, and 62 ± 4.1, respectively, during the study period (Figure 2a,b).

The nutrient composition of dietary ingredients and whole diets is presented in Table 2. The maize silage presented relatively high contents of DM (350 g/kg) and starch (381 g/kg, DM basis). As expected, the nutrient composition of concentrate mixtures was very similar, and that of the whole diets reflected the composition of the individual ingredients.

### 3.1. Lactation Performance

The DMI and yield of milk were, respectively, 1 kg/day (*p* = 0.038) and 2.7 kg/day (*p* = 0.002) higher for Yeast compared with that for Control cows (Table 3). However, energy-corrected milk (ECM) was similar (*p* = 0.148) for both treatments. Although not significant (*p* = 0.119), the reduction in milk fat concentration when cows were fed the Yeast diet could explain the lack of impact of treatment on ECM. Treatment had no impact on milk components, although tendencies were observed for an increase in milk protein concentration (*p* = 0.062) and a decrease in milk lactose concentration (*p* = 0.080) with live yeast supplementation.

### 3.2. Relationships Between Environmental Parameters and Animal Performance

The relationship between daily thermal amplitude, maximum, minimum, and mean temperatures, THI1 and THI2, and diet varied for each performance parameter (Table 4). Although different regression coefficients were found, both DMI and milk yield were influenced by all environmental parameters studied. Similarly, different regression coefficients were observed for ECM, milk protein, and fat production, but all these parameters were influenced by thermal amplitude, maximum, minimum, and mean temperatures, and mean THI1 and THI2.

The LRT showed that daily mean temperature and mean THI2 were the regression variables that best explained the variation in DMI and milk yield, respectively. Energy-corrected milk was best fitted with daily thermal amplitude and mean temperature, whereas daily thermal amplitude was the best predictor for milk protein production and daily minimum temperature for milk fat production.

**Figure 3:**
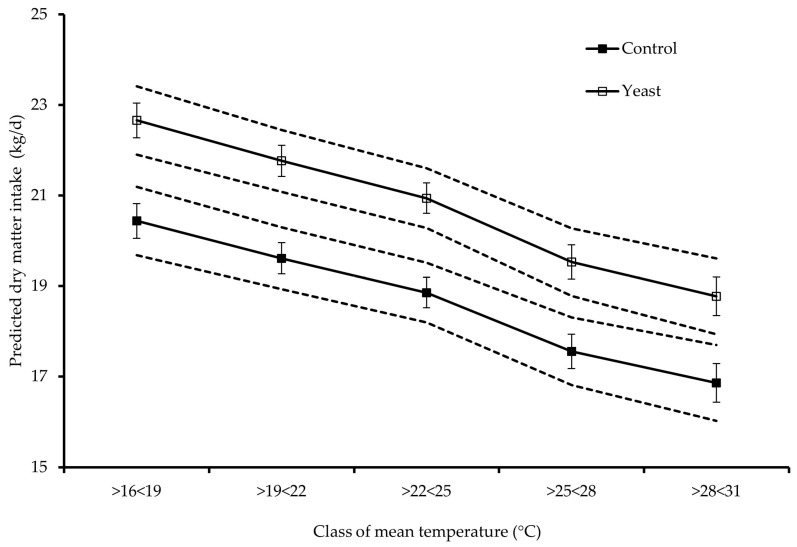
Relationship between classes of mean temperature and predicted dry matter intake (DMI). Dashed lines: upper and lower 95% confidence intervals for the predicted values in each diet. Vertical bars: standard error. Diets are named according to the inclusion of a live yeast culture based on *Saccharomyces cerevisae* strain 1026, respectively, without for Control or with live yeast supplementation for Yeast.

**Figure 4:**
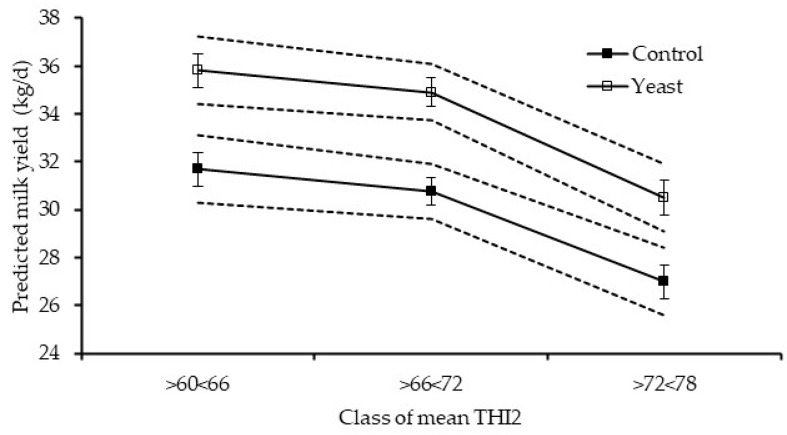
Relationship between classes of mean thermal humidity indices calculated according to ([35]; THI2) and predicted milk yield. Dashed lines: upper and lower 95% confidence intervals for the predicted values in each diet. Vertical bars: standard error. Diets are named according to the inclusion of a live yeast culture based on *Saccharomyces cerevisae* strain 1026, respectively, without for Control or with live yeast supplementation for Yeast.

Globally, Yeast promoted feed intake, milk yield, and composition for all classes of temperature and THIs studied. Regarding DMI, Yeast promoted an average increase of 2 kg DM per day for all classes of temperature and THIs studied except for mean THI1 (from 54 to 60), for which DMI was similar between treatments. Yeast promoted significantly higher milk yield than Control for all classes of temperature and THIs studied, averaging an increase of 4.0 kg of milk per day, except for the minimum temperature (from 24 to 29 °C), mean THI1 (from 78 to 84%), maximum THI1 (from 84 to 90%), and minimum THI2 (from 72 to 78%), for which milk production did not differ between diets. Figure 3 and Figure 4 present the plots of mean predicted values of DMI and milk yield against daily mean temperature and mean THI2 classes, respectively.

## 4. Discussion

In the present study, live yeast supplementation increased DMI and milk yield. However, as earlier stated, effect of the addition of cultures of *S. cerevisae* on dairy cow performance during summer conditions was not consistent. These conflicting results might be partially explained by the different conditions of each study, namely with respect to the stage of lactation, diet characteristics, and average maximal temperature, relative humidity, and the THI, as well as the duration of the period for the adaptation of the rumen population to yeast supplementation.

The term heat stress may refer to the climate, climatic effects on cows, or productive or physiologic responses of cows [12]. Despite the variation found in the literature, a THI value below 68 is generally considered safe for healthy animals, mild discomfort is present with a THI ranging from 68 to 74, and noticeable decreases in performance are shown for a THI above 75 [42,43]; thus, during the present study, cows were subjected to different levels of heat stress. The different relationships herein obtained between temperature and the THI and productive parameters (linear, quadratic, and cubic) suggest that these environmental factors differently affect the outcome of diet on animal performance. Indeed, some studies show different influence of climatic variables on productive parameters. For example, West [12] showed that day minimum air temperature was the variable with the greatest influence on cow a.m. milk temperature, whereas day mean air temperature best explained cow p.m. milk temperature. Additionally, Kabuga et al. [44] stated that when compared with minimum and maximum temperatures, mean daily temperature had the greatest effect on milk yield and rectal temperature.

As expected, independently of the diet, DMI and milk yield decreased with the rise in temperature. The relationship between thermoregulation and voluntary feed intake is in the basis of feed intake thermostatic regulation theory [45]. Although short exposures may have little effect, high-producing dairy cows are more vulnerable, especially during persistent hot weather or acute heat loads imposed by heat waves [46]. Feed intake and milk yield are most likely influenced by changes in body temperature [47], especially in hot weather conditions and mid-lactation [48]. On the other hand, early-lactation cows rely on body stores and late-lactation cows have a decreased feed intake; thus, effects of hot weather conditions on DMI are less pronounced. The decrease in plasma growth hormone concentration and growth hormone secretion with hot temperatures may also play an integral role in the decline in productivity (35 °C; [49]).

Intake of DM and milk yield were higher for Yeast than they were for Control cows for almost all classes of temperature parameters. Daily maximum temperature differently related to DMI (quadratic) and milk yield (cubic), raising the question of whether the reduced nutrient intake is the primary reason for the lower milk yield under heat stress or the cumulative effects of heat stress on feed intake, metabolism, and the physiology of dairy cattle. According to the study of Rhoads et al. [50], decreased nutrient intake accounted for just 35% of the decreased milk yield due to heat stress, and slight changes in the somatotropic axis may have contributed to a portion of the remainder. Similarly, Wheelock et al. [51] found that decreased nutrient intake contributes for only 50% of heat stress-induced decreases in milk yield; the additional decrease in milk yield is probably explained by feed intake-independent shifts in postabsorptive glucose and lipid homeostasis. That is, cows under heat stress present reduced lipid mobilization from the adipose tissue and increased glucose utilization by peripheral tissues—the normal glucose-sparing mechanisms not being engaged by these cows to maximize milk yield. Along with decreased nutrient intake, cows under heat stress are thought to have increased maintenance costs (30% greater; [52,53]), suggesting that in addition to insufficient energy intake, there is a shift in priorities for postabsorptive energy utilization resulting in a rapid drop in milk synthesis.

Based on the LRT, the models that used daily temperature parameters as the regression coefficient better explained the variation in DMI, ECM, and milk components than models that used THI parameters. Mean THI2 was the best covariable to explain milk yield variation. Despite the THI being commonly used to evaluate heat stress, the results obtained suggest a more remarkable effect of temperature on the performance parameters measured excluding milk yield. These results indicate that, although temperature–humidity interactions contribute significantly to the dynamics of milk yield, other production parameters may be directly influenced in isolation by ambient temperature. Indeed, Bohmanova, Misztal [6] found that the response to heat stress in milk production followed a broken-stick function, meaning that milk yield remained constant until a threshold of heat stress was surpassed and then linearly declined with a decreasing THI.

In the present study, depending on the considered THI, the number of days with the maximum THI below 71 ranged from 9 to 17, and that with a THI from 72 to 79 ranged from 11 to 19, with only 7 days with a THI between 80 and 89. No days exceed a THI of 90. Therefore, the cows were mainly subjected to mild stress. Thermal humidity indices explain the variation in animal performance differently, which demonstrates the vulnerability of using a single index to characterize heat stress. Similarly, Bohmanova, Misztal [6] found differences in thresholds of heat stress among indices and between regions, highlighting that the assessment of heat stress cannot be isolated from each farm system’s context. Although the THI remains a standard tool for assessing thermal stress, its utility is limited by individual variation in breed, age, parity, and health status, underscoring the need for personalized mitigation strategies [54]. Recent advances in non-invasive sampling, sensor technologies, and machine learning are broadening the scope of heat stress assessment beyond threshold detection, enabling proactive and targeted interventions. In addition, most studies to date are short-term and conducted under controlled environments, limiting their applicability to real-world conditions. There is a critical need for long-term studies to better understand the cumulative and adaptive responses to heat stress [9]. While tools such as infrared thermography, rumen boluses, and behavior monitors hold promise, their widespread adoption is hampered by high costs, technical complexity, and lack of field validation [55,56]. As recently concluded by Singaravadivelan, Prasad [9], research should prioritize the development of low-cost, user-friendly, and scalable technologies, particularly for small- to medium-scale producers; the integration of underutilized molecular tools (e.g., gene expression, epigenetics, and metabolomics) with phenotypic data could accelerate the genetic selection of heat-tolerant animals; and predictive platforms incorporating historical performance, meteorological data, and individual animal profiles offer opportunities for real-time decision support and improved resilience. Further investigation into the interaction between heat stress and the rumen microbiome may offer novel strategies for mitigation [13]. Overall, advancing heat stress management in dairy systems requires a multidisciplinary, welfare-driven approach that combines technological innovation with inclusive research strategies to ensure sustainable and climate-resilient dairy production.

## 5. Conclusions

Dietary supplementation with a live yeast culture based on *S. cerevisiae* effectively mitigated the negative effects of heat stress on dairy cow performance, preserving milk yield under high environmental temperatures. Environmental factors affect animals differently. Daily mean temperature is the best explanatory variable for DMI, and the thermal humidity index based on dew point temperature is the best predictor of its effect on milk yield. These findings highlight the potential of yeast supplementation to enhance the metabolic and physiological resilience of lactating cows exposed to heat stress. However, the physiological mechanisms by which yeast supplementation affects tolerance to high environmental temperatures must be better understood. Optimizing yeast supplementation protocols on dairy farms requires large-scale experiments under diverse environmental conditions. These experiments should integrate physiological markers such as body temperature and respiratory rate, as well as endocrine and stress proxies. This will contribute to developing broader strategies for climate-resilient dairy management systems and will allow for maintaining productivity and animal welfare in the face of increasing heat stress challenges driven by climate change.

## Figures and Tables

**Table 1:** Ingredient composition of experimental diets.

	Diet ^1^	
Ingredient, g/kg DM	Control	Yeast
Maize silage	420.0	420.0
Ryegrass hay	80.0	80.0
Concentrate mixture ^2^		
Wheat grain	2.0	2.0
Maize grain	105.0	105.0
Wheat bran	7.5	7.5
Cassava meal	8.7	8.7
Soybean meal	175.0	174.8
Corn gluten feed	113.8	113.6
Palm kernel meal	11.3	11.2
Sunflower meal	11.7	11.7
Molasses	2.0	2.0
Calcium soaps ^3^	12.5	12.5
Cottonseed	25.0	25.0
Sodium bicarbonate	5.0	5.0
Calcium carbonate	11.0	11.0
Magnesium oxide	3.0	3.0
Salt	2.5	2.5
Mineral–vitamin premix ^4^	1.5	1.5
Urea	2.5	2.5
Yeast culture ^5^	-	0.5

^1^ Diets are named according to the inclusion of a live yeast culture based *on Saccharomyces cerevisae* strain 1026, respectively, without for Control and with live yeast supplementation for Yeast. ^2^ Concentrate mixtures were prepared in a single batch by Cooperativa Agrícola de Vila do Conde, CRL (Vila do Conde, Portugal). ^3^ Vetagri Alimentar SA (Cantanhede, Portugal). ^4^ Contents: 5,000,000 U.I./kg vitamin A; 833,333 U.I./kg vitamin D3; 20,000 mg/kg vitamin E; 667 mg/kg beta-carotene; 133 mg/kg Co; 6667 mg/kg Cu; 3333 mg/kg Fe; 600 mg/kg I; 33,333 mg/kg Mg; 20,000 mg/kg Mn; 133 mg/kg Se; 26,667 mg/kg Zn. ^5^ Yea-sacc^1026^, live yeast culture based on *Sacchoromyces cerevisae* strain 1026, Alltech, Sintra, Portugal.

**Table 2:** Nutrient composition of dietary ingredients and whole diets (g/kg dry matter, DM).

	Forage		Concentrate		Diet ^1^	
Item	Maize Silage	Ryegrass Hay	Control	Yeast	Control	Yeast
DM, g/kg	350	870	900	910	667	672
Ash	33	60	119	107	78	72
CP	75	53	239	238	154	155
EE	27	10	34	36	29	30
NDF	392	565	252	264	336	342
ADF	229	338	109	117	178	182
ADL	42	42	34	45	38	43
Starch	381	ND ^2^	188	180	254	250
Urea	ND	ND	6.7	7.0	3.4	3.5
Ca	2.3	4.1	19.5	18.7	11.0	10.6
P	1.5	1.9	5.6	6.1	3.6	3.8

^1^ Diets are named according to the inclusion of a live yeast culture based on *Saccharomyces cerevisae* strain 1026, respectively, without for Control or with live yeast supplementation for Yeast. ^2^ Not determined.

**Table 3:** Adjusted means for productive parameters of dairy cows.

	Diet ^1^		SEM	*p*
	Control	Yeast		
DMI, kg/d	19.5	20.5	0.23	0.038
Milk				
Yield, kg/d	30.7	33.4	0.27	0.002
ECM ^2^, kg/d	31.7	33.1	0.53	0.148
Protein, %	2.66	2.76	0.029	0.062
Protein, kg/d	0.82	0.88	0.020	0.136
Fat, %	4.76	4.46	0.094	0.119
Fat, kg/d	1.44	1.46	0.044	0.867
Lactose, %	4.80	4.73	0.023	0.080
Lactose, kg/d	1.48	1.56	0.037	0.213

^1^ Diets are named according to the inclusion of a live yeast culture based on *Saccharomyces cerevisae* strain 1026, respectively, without for Control or with live yeast supplementation for Yeast. ^2^ Energy-corrected milk was calculated as milk yield (kg/d) × [(38.3 × milk fat (g/kg) + 24.2 × milk protein (g/kg) + 16.54 × milk lactose (g/kg) + 20.7)/3140].

**Table 4:** Coefficient of determination (r^2^), model significance (*p*-value), and significant regression coefficients (linear, quadratic, and cubic, as appropriate) for each model of daily temperature or THIs within diets after adjustment for the other variables in the model.

Dependent Variable	Covariable in the Model ^1^	r^2^	*p*-Value	Treatment ^2^	Linear	Quadratic	Cubic
DMI, kg/d	AMP	0.66	<0.001	Control	10.02	−0.70	0.02
				Yeast	15.37	−1.08	0.02
	MAX	0.71	<0.001	Control	0.89	−0.02	
				Yeast	0.99	−0.02	
	MIN	0.70	<0.001	Control	3.26	−0.22	0.01
				Yeast	4.44	−0.29	0.01
	MEAN	0.72	<0.001	Control	−0.31		
				Yeast	−0.34		
	MAX THI1	0.69	<0.001	Control	−0.18		
				Yeast	−0.21		
	MEAN THI1	0.69	<0.001	Control	0.88	−0.01	
				Yeast	1.44	−0.01	
	MIN THI1	0.69	<0.001	Control	−0.21		
				Yeast	−0.23		
	MAX THI2	0.70	<0.001	Control	1.63	−0.01	
				Yeast	2.06	−0.01	
	MEAN THI2	0.72	<0.001	Control	1.51	−0.01	
				Yeast	2.22	−0.02	
	MIN THI2	0.70	<0.001	Control	31.2	−0.49	0.01
				Yeast	35.7	−0.56	0.01
Milk yield, Kg/d	AMP	0.83	<0.001	Control	1.58	−0.06	
				Yeast	1.98	−0.08	
	MAX	0.87	<0.001	Control	−32.1	1.10	−0.01
				Yeast	−29.1	1.00	−0.01
	MIN	0.87	<0.001	Control	9.25	−0.58	0.01
				Yeast	10.96	−0.69	0.01
	MEAN	0.87	<0.001	Control	12.79	−0.57	0.01
				Yeast	20.99	−0.93	0.01
	MAX THI1	0.86	<0.001	Control	1.91	−0.01	
				Yeast	2.55	−0.02	
	MEAN THI1	0.87	<0.001	Control	2.72	−0.02	
				Yeast	3.67	−0.03	
	MIN THI1	0.86	<0.001	Control	2.68	−0.02	
				Yeast	3.54	−0.03	
	MAX THI2	0.87	<0.001	Control	−61.98	0.82	−0.01
				Yeast	−60.68	0.81	−0.01
	MEAN THI2	0.87	<0.001	Control	3.33	−0.03	
				Yeast	4.48	−0.04	
	MIN THI2	0.86	<0.001	Control	82.46	−1.28	0.01
				Yeast	95.97	−1.49	0.01
ECM ^3^, Kg/d	AMP	0.83	<0.001	Control	8.08	−0.29	
				Yeast	9.12	−0.34	
	MAX	0.80	<0.001	Control	−38.09	1.30	−0.01
				Yeast	−58.77	2.01	−0.02
	MIN	0.80	<0.001	Control	−0.59		
				Yeast	−0.64		
	MEAN	0.79	<0.001	Control	−0.45		
				Yeast	−0.57		
	MEAN THI1	0.79	<0.001	Control	−0.33		
				Yeast	−0.36		
	MEAN THI2	0.79	<0.001	Control	−0.38		
				Yeast	−0.47		
Milk protein, kg/d	AMP	0.80	<0.001	Control	0.23	−0.01	
				Yeast	0.24	−0.01	
	MAX	0.79	<0.001	Control	−0.95	0.03	−0.01
				Yeast	−1.55	0.05	−0.01
	MIN	0.77	<0.001	Control	0.05	−0.01	
				Yeast	0.07	−0.01	
	MEAN	0.76	<0.001	Control	−0.02		
				Yeast	−0.02		
	MEAN THI1	0.75	<0.001	Control	−0.01		
				Yeast	−0.01		
	MEAN THI2	0.77	<0.001	Control	0.13	−0.01	
				Yeast	0.18	−0.01	
Milk fat, kg/d	AMP	0.70	<0.001	Control	0.34	−0.01	
				Yeast	0.42	−0.02	
	MAX	0.68	<0.001	Control	−1.43	0.05	−0.01
				Yeast	−3.14	0.11	−0.01
	MIN	0.67	<0.001	Control	−0.02		
				Yeast	−0.03		
	MEAN	0.66	<0.001	Control	−0.01		
				Yeast	−0.02		
	MEAN THI1	0.66	<0.001	Control	−0.01		
				Yeast	−0.01		
	MEAN THI2	0.66	<0.001	Control	−0.01		
				Yeast	−0.02		

^1^ AMP = thermal amplitude; MAX = maximum temperature; MIN = minimum temperature; MEAN = mean temperature; THI1 = thermal humidity index calculated according to [34]; THI2 = thermal humidity index calculated according to [35]. ^2^ Diets are named according to the inclusion of a live yeast culture based on *Saccharomyces cerevisae* strain 1026, respectively, without for Control or with live yeast supplementation for Yeast. ^3^ Energy-corrected milk.

## Data Availability

The original contributions presented in this study are included in the article. Further inquiries can be directed to the corresponding authors.

## Abbreviations

The following abbreviations are used in this manuscript:

ADF	Acid detergent fiber
ADL	Acid detergent lignin
CP	Crude protein
DIM	Days in milk
DM	Dry matter
DMI	Dry matter intake
ECM	Energy-corrected milk
EE	Ether extract
LRT	Likelihood ratio test
NDF	Neutral detergent fiber
SEM	Standard error of the mean
SNFs	Solids not fat
Tdp	Dew point temperature
THI	Thermal humidity index
TMR	Total mixed ration
Twb	Wet bulb temperature
VFA	Volatile fatty acid
